# Comparison of HIV self-test distribution modalities to reduce HIV transmission and burden in western Kenya: a mathematical modelling study

**DOI:** 10.1136/bmjopen-2025-102999

**Published:** 2025-07-30

**Authors:** Hae-Young Kim, Ingrid Eshun‐Wilson, Daniel Bridenbecker, Cheryl Johnson, Christine Kisia, Jonah Onentiah Magare, Elvin H Geng, Anna Bershteyn

**Affiliations:** 1 New York University Grossman School of Medicine, New York City, New York, USA; 2 Washington University in St Louis, St. Louis, Missouri, USA; 3 Gates Foundation, Seattle, Washington, USA; 4 World Health Organization, Geneva, Switzerland; 5 World Health Organization, Nairobi, Kenya; 6 National AIDS and STIs Control Program (NASCOP), Nairobi, Kenya

**Keywords:** HIV & AIDS, Epidemiology, Africa South of the Sahara

## Abstract

**Abstract:**

**Objectives:**

To compare the impact of different HIV self-testing (HIVST) distribution modalities on population-level HIV outcomes.

**Design:**

Mathematical modelling study.

**Setting:**

Six counties in western Kenya.

**Methods:**

We projected population-level HIV outcomes among individuals aged 15+over 30 years (2022–2052) using EMOD-HIV, an agent-based network transmission model calibrated to the HIV epidemic in western Kenya. We simulated the impact of three HIVST distribution modalities: (1) secondary distribution to male partners via women who attend antenatal care visits (‘ANC’); (2) secondary distribution to partners of individuals newly diagnosed with HIV at health facilities (‘partner services’); and (3) distribution to any individuals attending outpatient clinics (‘outpatient’). We informed our model assumptions on HIV testing uptake for each HIVST distribution modality using the estimates from a meta-analysis of randomised clinical trials published between 2006 and 2019 and compared the outcomes for each HIVST distribution modality to standard testing without HIVST.

**Outcomes measures:**

The number of HIV tests performed (HIVST and non-HIVST), HIV diagnoses, HIV infections, and HIV-related deaths.

**Results:**

With standard testing alone, the average number of HIV tests was 4.69 million per year, amounting to 81.0 tests per 100 adults. The average number of tests per year increased by 2.9% with ANC, 0.6% with partner services, and 23.7% with outpatient distribution of HIVST. Compared with standard testing alone, partner services with HIVST will avert the largest number of new HIV infections (10.2%, 95% CI 9.9% to 10.5%), followed by outpatient distribution (8.5%, 95% CI 8.2% to 8.7%) and ANC (6.1%, 95% CI 5.8% to 6.3%). Compared with standard testing, the number of HIVST needed per one additional HIV infection averted was 216 with ANC, 17 with partner services and 2009 with outpatient, while the number of HIVST per one additional HIV-related death averted was 364 with ANC, 17 with partner services and 3851 with outpatient.

**Conclusions:**

Secondary distribution of HIVST to partners of individuals newly diagnosed with HIV could prevent the most HIV infections and HIV-related deaths. HIVST can be an important strategy to improve uptake of testing and long-term population-level health effects.

**STRENGTHS AND LIMITATIONS OF THIS STUDY**

- We estimated the effect of HIV self-testing (HIVST), an effective and acceptable method of HIV diagnosis, on population-level HIV outcomes over a 30-year period.
- We used an agent-based sexual network transmission model that has been previously validated and calibrated to the HIV epidemic in six counties in western Kenya, ensuring the model’s accuracy and relevance to the region. These counties are located along Lake Victoria and have the highest HIV prevalence in Kenya.
- Our model was informed by meta-analysis estimates from the randomised clinical trials in Eastern and Southern Africa which examined the effects of three HIVST distribution strategies on HIVST uptake and linkage to care.
- We did not evaluate potential services for individuals with a negative self-test and the use of HIVST as part of pre-exposure or postexposure prophylaxis implementation. We did not evaluate the broad range of HIVST distribution strategies, such as those in the private sector, workplaces or the broader community.

## Introduction

 Regions hard-hit by the HIV/AIDS pandemic have made significant progress towards the World Health Organization’s (WHO) targets to eliminate AIDS by 2030.^1^ However, in many regions, the largest remaining gap is diagnosing people living with HIV who are unware of their status. According to the latest population-based HIV impact assessment conducted in Kenya, 80% of people living with HIV were aware of their HIV status, while 96% of people diagnosed with HIV were on treatment and 91% of those on treatment were achieving viral load suppression in 2018.^2^ Gaps in diagnosis are often larger in men than in women: only 79% of men living with HIV were diagnosed, compared with 90% of women in western Kenya. More effective testing approaches are needed to close these gaps in HIV diagnosis.

Since 2016, the WHO has recommended HIV self-testing (HIVST) to improve access to testing. HIVST allows individuals to test themselves when and where they want through a broad range of options such as at home, in a facility, or another place of their choosing.^3 4^ Numerous studies have shown that HIVST is acceptable across various populations including young people,^5^ men,^6 7^ partners,^8^ key populations,^9 10^ pharmacy clients^11^ and those using pre-exposure or postexposure prophylaxis (PrEP or PEP).^12^ Several randomised clinical trials (RCTs) have also investigated different ways to distribute HIVST and their impact. For example, distributing HIVST through women attending antenatal care (ANC) visits substantially increased HIV testing uptake among male partners in Kenya^8^ and Malawi^13^ as well as new HIV diagnosis in Malawi.^13^ Distributing HIVST through HIV-positive clients attending facilities increased HIV testing and new HIV diagnosis among their sexual partners in Malawi.^14^

Recognising the value of HIVST, Kenya pioneered the approach by establishing the world’s first HIV self-testing policy.^15^ Since then, there has been considerable natinoal scale-up, with wide-scale implementation across the country. According to global reporting to WHO, Kenya distributed more than half a million HIVST kits in 2023. While access to low-cost quality-assured products, such as those available for US$1, has improved, opportunities for further scale-up remain, and the need and demand for HIVST in Kenya and the broader region are still largely unmet.^16^ For example, of adults who have ever been tested for HIV, only 4% in Kenya^17^ and 1.2% in Zimbabwe and Malawi^18^ reported having ever taken an HIV self-test. Moreover, clinics and pharmacies also face frequent stock-outs and limited supplies, while the national programme faces challenges in accurately quantifying and distributing kits to those in need of HIVST.^19^ Therefore, optimising the distribution of HIVST is critical to maximise its impact, effectively address the HIV epidemic, and support national planning at the government level.

In this study, we incorporated data from randomised controlled trials (RCTs) on HIVST uptake and linkage to care into a previously validated agent-based network model of the HIV epidemic in western Kenya. Using this model, we compared the impact and efficiency (ie, impact per HIVST used) of different HIVST distribution strategies. This study may help to inform the best use of currently available HIVST supply, while also informing potential strategies for the expansion of HIVST delivery if the supply were to increase.

## Methods

### Model structure

We simulated the effect of different HIVST distribution modalities using EMOD-HIV, which is part of the EMOD disease transmission modelling framework.^20 21^ EMOD-HIV is an open-source, agent-based model that incorporates vertical and sexual HIV transmission based on an age/sex-structured relationship network.^22 23^ It also incorporates within-individual dynamics of HIV disease progression, the effects of treatment and utilisation of HIV testing, care and prevention along a care continuum, as described in detail previously.^20^

Within the model, baseline HIV testing modalities include voluntary or provider-initiated testing at healthcare facilities, testing of pregnant women during ANC visits, and symptomatic testing for individuals who progress to advanced HIV disease. For voluntary or provider-initiated testing at health facilities, individuals uptake HIV testing on their sexual debut, with the probability following a sigmoid function that varies by calendar year (as detailed in the *Model assumptions and validation section*). Individuals who do not undergo HIV testing at sexual debut are assigned a random probability of testing once a year thereafter. After an individual completes his or her initial HIV test, the frequency of repeat HIV testing remains annual, with a random, exponentially distributed time between repeat tests.

### Model calibration

EMOD-HIV has been calibrated to the HIV epidemic in six counties in western Kenya comprising the former province of Nyanza, namely Homa Bay, Kisii, Kisumu, Nyamira, Migori and Siaya.^24 25^ This region has the highest HIV prevalence in Kenya, with one in four adults living with HIV in Homa Bay and Siaya counties, and one in five adults living with HIV in Kisumu county, in 2018.^2^ Model calibration methods, resulting epidemic curves, and associated model parameter values and their distributions are publicly available.^24 25^ Briefly, the model was fit to age-stratified, sex-stratified and county-stratified HIV prevalence estimates from 2003^26^ and 2008–2009^27^ Demographic and Health Surveys, 2007^28^ and 2012^29^ AIDS Indicator Surveys and the Kenya Population-based HIV Impact Assessment (KENPHIA) 2018,^2^ numbers of people receiving treatment reported annually by the Kenya Ministry of Health, and population structure and density estimated in the Kenya Population and Housing Census.^30^ The model fit for HIV prevalence and HIV incidence is shown in online supplemental figures S1 and S2. The model uses annual estimates of age-specific fertility rates and age-specific and sex-specific non-HIV mortality rates from the United Nations Population Division to generate a simulated population into which HIV is introduced in 1982. Model parameters related to sexual behaviour were modified to fit the above-described data using a stochastic optimisation algorithm known as parallel simultaneous perturbation optimisation,^31^ which maximises the model’s posterior probability of fitting the available data while accounting for the uncertainty of each estimate. We selected a set of 250 best-fit model trajectories using roulette resampling in proportion to likelihood.

### Model assumptions and validation

For voluntary or provider-initiated testing at health facilities, we assumed that the probability of HIV testing uptake at sexual debut follows a sigmoid function, increasing from 0% to 5% with an inflection point in 2005. The probability of HIV testing uptake for annual repeat testing after sexual debut is also assumed to increase sigmoidally from 0% to 15%, with an inflection point in 2006, and then continue to increase based on the reported data on annual HIV testing and overall HIV testing history. In our model simulations, the average percentage of adults aged ≥15 years who report having ever tested for HIV was 80.3% in 2018, increasing to 82.1% in 2021. This is comparable to the reported figure of 78% from the KENPHIA 2018 survey.^2^ Additionally, our simulations estimated that the overall percentage of individuals aged 15–49 who received an HIV test in the past 12 months was 47.4% (48.6% in women and 46.4% in men) in 2018. This was slightly lower than the reported figures in the six counties, which ranged from 54.1% to 76.2% in women and 44% to 64% in men in 2018.^2^

The probability of HIV testing at 12 weeks of pregnancy during ANC visits was assumed to increase sigmoidally from 0% in 1990 to 98% in 2011 based on the Kenya AIDS indicator surveys, in which 97.3% of women aged 15–49 years who delivered reported attending at least one ANC visit and of these, 96.0% knew their HIV status.^2 28 29^ Similarly, the probability of symptom-driven HIV testing was assumed to increase sigmoidally from 0% in 1990 to 95% in 2016. This assumption was based on a survey conducted in coastal Kenya from 2017 to 2019, which found that 93.1% of individuals who presented to clinics with symptoms of acute infectious illness and were offered HIV testing accepted it.^32^ Lastly, we assumed that the sensitivity and specificity of rapid HIV tests are both 100%.

### Model scenarios: HIVST distribution modalities

Based on a meta-analysis,^33^ we identified three HIVST distribution modalities with sufficient evidence to estimate the uptake of HIVST and its effect on HIV diagnosis. These modalities included: (1) secondary distribution through women who attend ANC visits to their male partners (‘ANC’); (2) secondary distribution through individuals newly diagnosed with HIV to their current sexual partners (‘partner services’) and (3) distribution of HIVST at outpatient facilities (‘outpatient’).

We assumed that secondary distribution of HIVST via the ANC and partner services would result in HIVST provision to up to two current sexual partners. We assumed that outpatient distribution would result in HIVST provision to adults aged 15 and 49 years. For all modalities, we assumed HIVST distribution would begin in 2022 and continue through 2051, the end of the period of analysis.

Effect sizes for the uptake of HIVST were informed by the meta-analysis results,^33^ comparing the modalities to the proportion of HIV testing uptake in the respective groups as of 2021. In scenarios of secondary distribution via ANC women and partner services, we assumed that HIVST kits and referral letters are provided to partners for confirmatory testing at healthcare facilities.^13 34^ In the outpatient modality, we assumed that HIVST kits are distributed at outpatient waiting spaces where self-testing and result interpretation would occur in private spaces.^35^

HIVST kits used by each distribution strategy and their effects on the proportion of the population tested for HIV were configured using the data from HIVST RCTs identified in a recent systematic review.^33^ We included four RCTs of ANC distribution^8 13 34 36^ (two in Kenya and two in Malawi); two RCTs of distribution via partner services in Malawi^13 34^ and three RCTs of outpatient modality^35 37 38^ (two in Kenya and one in Malawi). All RCTs measured the proportion of population receiving HIVST in the intervention arms and standard-of-care testing both in the intervention and control arms.

Across these RCTs, the overall rate ratio for HIV testing was 2.63 (95% CI 1.81 to 3.82) for ANC distribution, 2.03 (95% CI 1.01 to 4.09) for the partner services, and 2.38 (95% CI 0.97 to 5.83) for outpatient distribution, compared with standard testing. When combined with HIV testing rates in our model, the corresponding odds ratios (ORs) for HIV testing were 13.32 (95% CI 2.15 to 82.73), 4.15 (95% CI 0.63 to 27.81) and 4.17 (95% CI 1.05 to 16.60) in ANC, partner services and outpatient modalities, respectively.

In the meta-analysis of RCTs which compared linkage to HIV treatment or care for those diagnosed using HIVST versus standard testing, the risk ratios (RRs) for linkage to care were 0.94 (95% CI 0.58 to 1.53) for ANC distribution, 1.07 (95% CI 0.89 to 1.28) for partner services, and 0.84 (95% CI 0.55 to 1.30) for outpatient distribution of HIVST. Overall, the linkage rate with HIVST was comparable to that of standard testing (RR 0.95, 95% CI 0.79 to 1.13).^33^ Given the uncertainty of available linkage estimates and heterogeneity across modalities, we conservatively assumed that linkage to care was 5% lower for individuals receiving HIVST compared with those undergoing standard testing, regardless of the distribution modality.

### Model outcomes

Over a 30-year time period, we calculated the total number of HIV tests and HIVST, new HIV infections, HIV incidence and HIV-related deaths, and individuals on antiretroviral treamtent (ART) among those aged 15+ years (table 1). Model outcomes, disaggregated by males and females, are presented in the Supplementary Materials (online supplemental table S1). The average number of tests per year was calculated as the total number of tests over the 30-year time period divided by 30 years. We also analysed the number of HIVST needed per additional HIV infection averted or HIV-related death averted when using different distribution modalities.

**Table 1:** Estimated number of HIV tests and outcomes by HIVST distribution modality*

	SoC	Scenario 1: ANC	Scenario 2: partner services	Scenario 3: outpatient
HIV tests per year (1000s)	4688 (4683 to 4693)	4824 (4820 to 4829)	4717 (4713 to 4722)	5801 (5797 to 5804)
Non-HIVST (1000s)	4688 (4683 to 4693)	4738 (4734 to 4743)	4706 (4702 to 4710)	4686 (4685 to 4687)
HIVST (1000s)	–	86.0 (85.6 to 86.3)	11.4 (11.2 to 11.6)	1115 (1113 to 1117)
HIV infections				
New HIV infections per year (1000s)	6.5 (6.4 to 6.6)	6.1 (6 to 6.2)	5.8 (5.7 to 5.9)	6.0 (5.9 to 6.1)
Cumulative new HIV infections (1000s)†	195.6 (192 to 199.1)	183.7 (180.4 to 186.9)	175.4 (172.4 to 178.5)	178.9 (175.7 to 182.1)
HIV-related deaths				
HIV-related deaths per year (1000s)	7.5 (7.4 to 7.6)	7.2 (7.2 to 7.3)	6.8 (6.7 to 6.9)	7.2 (7.1 to 7.3)
Cumulative HIV-related deaths (1000s)†	224.4 (221.5 to 227.3)	217.3 (214.5 to 220.1)	203.9 (201.4 to 206.5)	215.7 (212.9 to 218.5)
Antiretroviral treamtent (ART)				
Number of people on ART per year (1000s)	341.1 (336.5 to 345.6)	341.0 (336.4 to 345.5)	347.2 (342.5 to 351.8)	342.7 (338.1 to 347.3)
Cumulative person-years on ART (1000s)†	10 233 (10 094 to 10 368)	10 230 (10 091 to 10 365)	10 415 (10 275 to 10 553)	10 282 (10 143 to 10 418)

* 95% CIs are shown in parentheses.

† Cumulative HIV infections, HIV-related deaths, and person-years on ART are over the 30 years of simulation period (2022–2051).

ANC, antenatal care; HIVST, HIV self-testing; SoC, standard-of-care.

The reported results represent the means of 250 best-fit model trajectories derived from the calibration process. The 95% CIs were estimated using 10 000 bootstrap samples generated using R package boot. Reporting of this study followed the updated Consolidated Health Economic Evaluation Reporting Standards 2022 guideline.^39^

### Sensitivity analyses

In one-way sensitivity analyses, we varied the effects of HIVST on uptake using the 95% CIs of the effect estimates from the meta-analysis. Additionally, we limited the time horizon of the analysis to 10 years to assess the relatively short-term policy impact, as HIV testing and treatment methods may change over the next 30 years (online supplemental tables S2 and S3).

### Patient and public involvement

No patients or members of the public were involved in the design, conduct, or reporting of this research.

## Results

**Figure 1:**
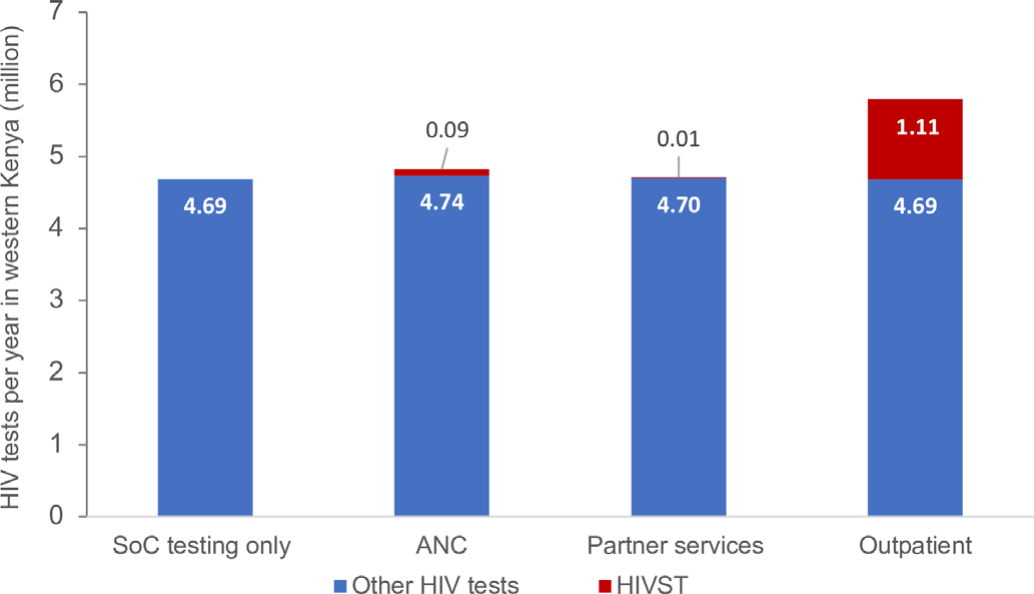
Number of HIV tests per year by HIVST distribution modalities. ANC, secondary distribution through women who attended antenatal care visits to their male partners; Partner services, secondary distribution through individuals newly diagnosed with HIV to their current sexual partners; Outpatient, distribution of HIVST at outpatient facilities. ANC, antenatal care; HIVST, HIV self-testing; SoC, standard of care.

With standard testing alone (no HIVST), the average number of HIV tests among those aged 15+ years between 2022 and 2052 was 4.69 million per year, amounting to 81.0 tests per 100 adults (table 1). In scenarios including HIVST, the average number of tests per year increased by 2.9% (4.82 million) with ANC distribution, 0.6% (4.72 million) with partner services and 23.7% (5.80 million) with outpatient distribution. Of the total number of HIV tests, the proportion comprised by HIVST was 1.8% for ANC distribution, 0.2% for partner services and 19.2% for outpatient distribution (figure 1).

Estimated HIV incidence in 2052 was 0.049% (95% CI 0.048% to 0.050%) in the standard testing scenario. HIVST was projected to reduce HIV incidence in 2052 to 0.046% (95% CI 0.044% to 0.047%) with ANC distribution, 0.042% (95% CI 0.041% to 0.043%) with partner services and 0.045% (95% CI 0.045% to 0.046%) with outpatient distribution.

**Figure 2:**
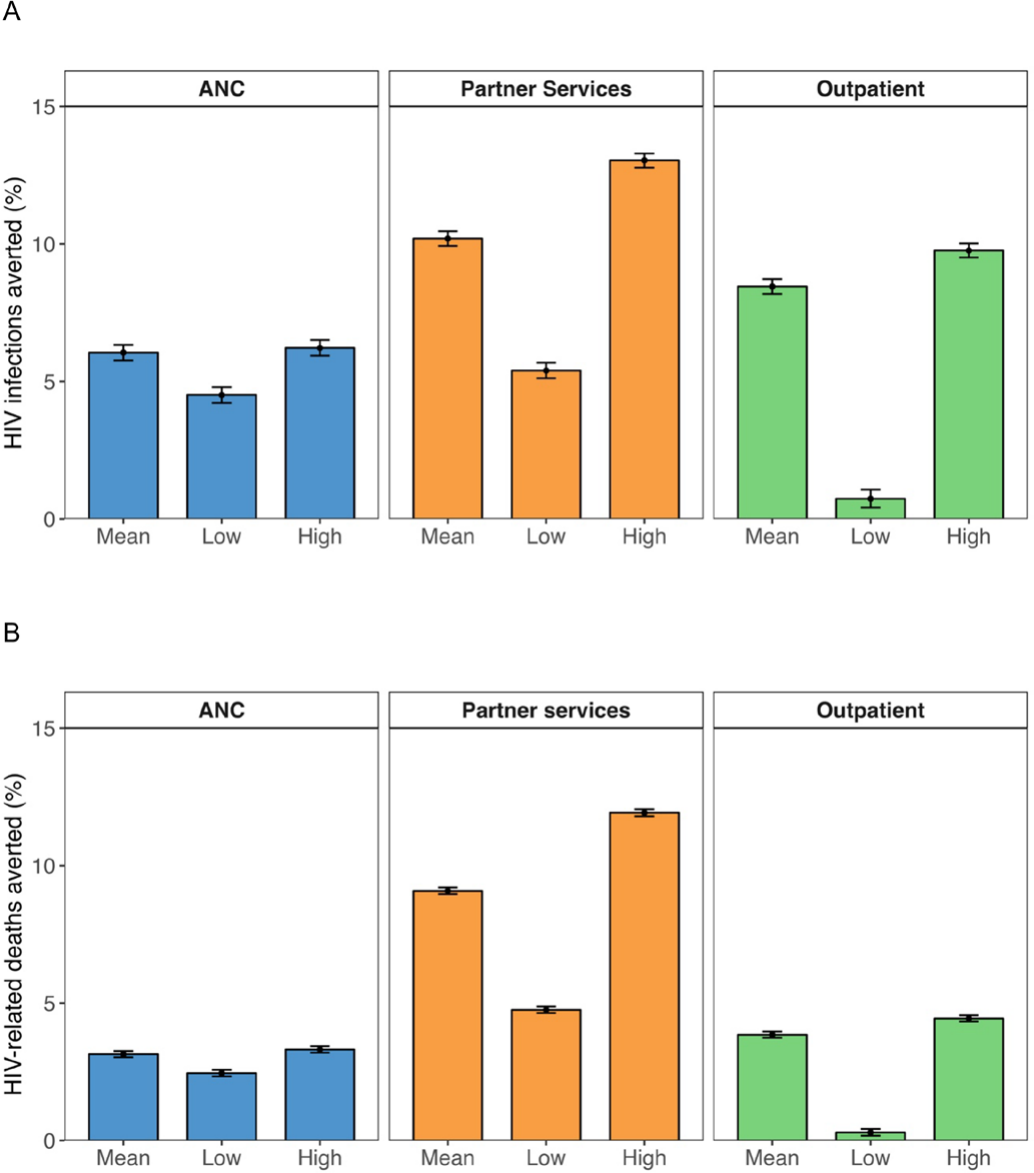
(**A**) HIV infections averted and (**B**) HIV-related deaths averted between 2022 and 2051 by different HIVST distribution modalities, compared with the standard of care testing only. For each distribution modality, ‘mean’ represents the results assuming the mean effect of HIVST testing uptake from the meta-analysis, while ‘low’ and ‘high’ represent the results assuming the lower and upper bounds of the 95% CI for the effects of HIVST testing uptake in the meta-analysis, respectively, while holding all other parameter values constant. The error bars represent the 95% CI from bootstrap analysis of 250 simulations. ANC, secondary distribution through women who attended antenatal care visits to their male partners; Partner services, secondary distribution through individuals newly diagnosed with HIV to their current sexual partners; Outpatient, distribution of HIVST at outpatient facilities. ANC, antenatal care; HIVST, HIV self-testing.

The number of new HIV infections averted over the period of simulated distribution (2022–2052) was highest for the partner services at 10.2% (95% CI 9.9% to 10.5%) of new HIV infections (n=20 131 (95% CI 19 410 to 20 825)), followed by the outpatient modality (8.5% (95% CI 8.2% to 8.7%); n=16 645 (95% CI 16 005 to 17 286)) and ANC (6.1% (95% CI 5.8% to 6.3%); n=11 912 (95% CI 11 295 to 12 505)). Similarly, the partner services averted the largest number of HIV-related deaths, 9.1% (95% CI 9.0% to 9.2%) (n=20 481 (95% CI 20 014 to 20 927)), followed by the outpatient modality (3.8% (95% CI 3.7% to 4.0%); n=8686 (95% CI 8392 to 8988)) and ANC (3.1% (95% CI 3.0% to 3.3%); n=7080 (95% CI 6801 to 7363)) (figure 2).

The number of HIVST needed to avert one additional HIV infection was lowest for partner services at 17 tests per infection averted, followed by ANC distribution at 216 tests per infection averted, and highest for outpatient distribution at 2009 tests per infection averted. The number of HIVST needed to avert one additional HIV-related death was also lowest for partner services at 17 tests per death averted, followed by ANC distribution at 364 tests per death averted, and highest for outpatient distribution at 3851 tests per death averted.

**Figure 3:**
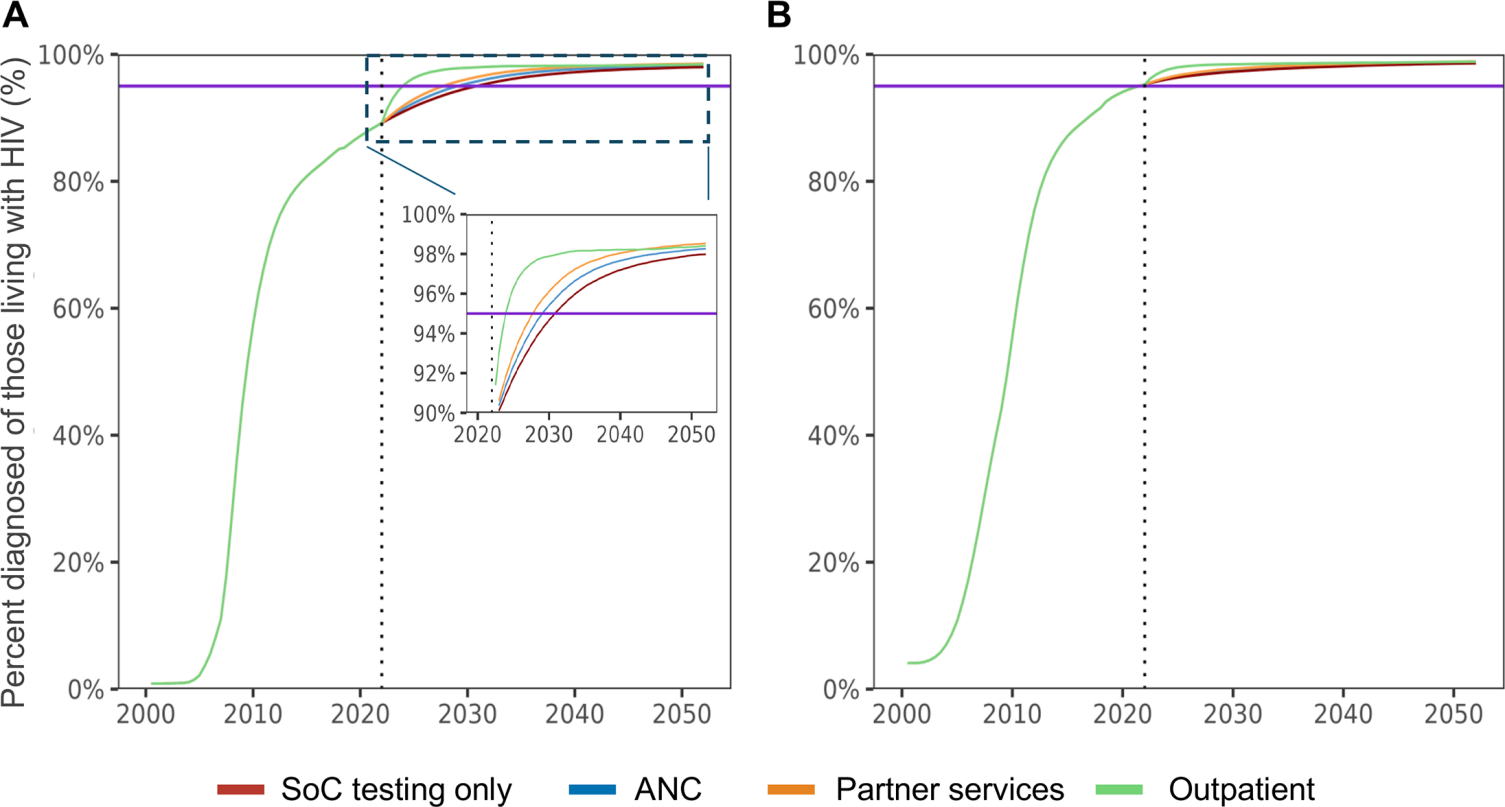
Estimated percentages of individuals diagnosed with HIV among those living with HIV by HIVST distribution modalities: (**A**) males and (**B**) females. The purple line indicates 95% (ie, the ‘First 95’ of the UNAIDS 95-95-95 targets). The dotted line indicates the year when the simulation starts (2022). ANC, secondary distribution through women who attended antenatal care visits to their male partners; Partner services, secondary distribution through individuals newly diagnosed with HIV to their current sexual partners; Outpatient, distribution of HIVST at outpatient facilities. ANC, antenatal care; HIVST, HIV self-testing; SoC, standard of care.

With standard testing alone, it will take until 2031 to diagnose >95% of men living with HIV (‘First 95’). With HIVST, the First 95 would be reached by 2025 with outpatient distribution, 2028 with partner services and 2029 with ANC distribution (figure 3). Our model projected that females have already reached the First 95 by 2023 regardless of distribution strategies.

HIVST can increase the need for ART by diagnosing individuals earlier in the course of HIV infection, yet it can also reduce the need for ART by averting new HIV infections. Person-years on ART increased in earlier years of HIVST distribution and decreased in later years due to the accruing effect of averted HIV infections (online supplemental figure S3). Over 2022–2052, the effect of HIVST on the number of person-years on ART was −0.03% (95% CI −1.38% to 1.29%) with ANC distribution but 1.78% (95% CI 0.41% to 3.13%) with partner services and 0.48% (95% CI −0.87% to 1.81%) with outpatient distribution. The year when the number of person-years on ART became lower compared with that in the standard testing scenario was 2037 with ANC distribution, 2060 with partner services and 2037 with outpatient facility-based distribution.

## Discussion

We simulated the effect of three different HIVST distribution modalities on uptake of HIV testing and long-term HIV epidemic trajectories in western Kenya. The outpatient modality distributed by far the largest number of HIV tests, increasing the total number of HIV tests by nearly one-quarter. However, the partner services averted the largest number of new HIV infections and HIV-related deaths, both per test distributed and overall. Our network model estimated that it would take just 17 tests via the partner services to avert one HIV-related death, and similarly 17 tests to avert one HIV infection. The partner services increased the number of person-years on ART slightly, but it will become lower than standard testing by 2060.

Our findings suggest that the partner services would maximise the impact of a limited HIVST supply. At most, HIVST distribution via the partner services could avert as many as 10% of HIV infections and 9% of HIV deaths over the 30 years, even after accounting for the potential of a slightly lower or delayed linkage rate after a positive HIVST compared with that in standard facility-based testing. After partner services needs are satisfied, the next-most-efficient modality for HIVST distribution would be the secondary distribution via ANC client to their partners, followed by distribution to outpatients. Our findings further suggest that the partner services would initially increase the number of person-years on ART by testing and diagnosing more people with HIV but will eventually reduce those on ART by averting new HIV infections. Opportunities to consider building on these findings to expand network-based testing more broadly and integration with HIV/syphilis dual self-tests should also be considered to further increase impact over time. Additionally, our analysis only considers the impact of HIVST among people living with HIV, but does not consider potential benefits for scaling up PrEP or PEP services which would further enhance its impact and contribution to achieving national and global goals.

Our study aligns with current WHO guidelines and national guidelines in many sub-Saharan African countries recommending HIVST distribution via the partner services. HIV prevalence among partners of patients newly diagnosed who received HIVST has been determined to be as high as 15%,^34^ as compared with only 3% HIV prevalence among outpatients in similar settings.^35^ Moreover, partner services and ANC secondary distribution disproportionately reach men, helping to close the gender gap in HIV diagnosis. In a study of the effect of HIVST distribution to partners of patients newly diagnosed in Malawi, 72.7% of new index patients were females who distributed HIVST kits to their male partners.^34^ Men are currently underdiagnosed relative to women, a gap that has been attributed to multiple barriers including anticipated stigma and discrimination, wait time and low perception of risk.^40^ A recent systematic review of 37 studies on HIVST uptake and intervention strategies in sub-Saharan Africa (SSA) demonstrated that HIVST was highly acceptable among men across many countries.^6^ The main facilitators were ease of use, privacy and efficiency, compared with health facility-based testing.^41^,^43^ HIVST distribution via partner services could dovetail with couples-oriented interventions such as assisted partner notifications to reduce downstream barriers to confirmatory testing and linkage to care.^16^

A study in western Kenya reported that 94% of participants in the general population found HIVST to be acceptable.^44^ Our results suggest that scaling up HIVST programmes in countries with suboptimal testing uptake could be a valuable strategy. Additionally, implementing options such as facility-based distribution yielded the largest number of additional HIV tests to the population. In Malawi,^45^ facility-based HIVST led to nearly four times the uptake of HIV testing in the outpatient population compared with only standard provider-initiated testing. It allowed more outpatients to complete tests in a shorter timeframe with the assistance of a single dedicated facility worker, whereas standard provider-initiated testing was relatively slower due to an insufficient number of available health staff and the lack of private space and time needed for one-on-one testing.^45^ Our analysis focused on HIVST supply constraints, finding that distributing HIVST to outpatients would require significantly more tests to yield a similar impact as secondary distribution. However, health systems with sufficient HIVST supply and with healthcare workforce constraints may find efficiencies in outpatient distribution. In Kenya, healthcare providers reported that HIVST distribution among outpatient populations reduced their workload, allowing them to focus on other clinical responsibilities.^46^ This is critical for supporting primary healthcare systems and integration efforts, which are increasingly demanding health worker time and being prioritised globally due to growing needs such as non-communicable diseases, testing needs driven by triple elimination initiatives^47^ and the growing burden of curable STIs and antimicrobial resistance.

Through implementation projects like the HIV Self-Testing Africa Initiative,^48^ a growing number of countries in SSA are incorporating HIVST as part of their HIV testing services. While rates may vary depending on the delivery modalities and provision of assistance during HIVST, recent meta-analyses and systematic reviews have shown that linkage to confirmatory testing and care is generally high and comparable to standard testing.^33 49^ Furthermore, HIVST presents significant opportunities to expand access to prevention services, including PrEP and PEP.^50^ It can facilitate the initiation, continuation or reinitiation of PrEP services both at health facilities^51^ and remotely, such as through community-based pharmacies^52 53^ and mobile platforms,^54^ and prevent new infections among those currently unreached by prevention services.^55^ Based on this evidence, WHO recommends using HIVST for initiating and continuing PrEP and PEP.

Our study has several limitations. First, we did not consider potential services for individuals with a negative self-test, such as accessing PEP or initiation or continuation of PrEP. Second, we did not evaluate the costs or cost-effectiveness of HIVST, as implementation costs were not available for all distribution modalities. A recent modelling study in South Africa reported that secondary distribution to partners of ART patients had the greatest epidemiological impact but was the least cost-effective strategy, while distribution of HIVST at workplaces would be cost saving but have a moderate impact on averting HIV infections.^56^ In the context of PrEP, a recent study showed using HIVST over standard testing in Kenya was more affordable and less costly.^57^ Also, the scale-up of HIVST distribution can be complex and depends on many other factors, such as supply chain, healthcare infrastructure and public awareness, which have not been explicitly modelled in our analyses.

Looking ahead, it is critical to evaluate the role of HIVST in addressing gaps in delivery of timely HIV prevention services and diagnosis, while achieving more efficient benefits. Expanding the integration of HIVST and delivery systems beyond traditional HIV service points—such as worksites, private pharmacies and mobile platforms—could further enhance accessibility, uptake and costs. HIVST could also facilitate earlier diagnoses when offered a sufficiently high volume. While most cost evaluation or cost-effectiveness analyses of HIVST have been conducted from a health system perspective, assessing cost-effectiveness from both end-user and societal perspectives will be valuable. Lastly, future studies could consider budget impact analysis to determine the proportion of HIVST within overall HIV programmes and health system budgets, offering insights into the implications of incorporating HIVST for optimal resource allocation.

## Conclusions

HIVST has the potential to substantially increase HIV testing and reduce new infections and deaths. While the distribution of HIVST to outpatients could have the largest impact on HIV testing uptake, secondary distribution of HIVST by individuals newly diagnosed with HIV to their sexual partners would avert more infections and deaths, both overall and per test distributed. Expanding HIVST distribution while incorporating innovative approaches to follow-up and linkage to both prevention and care is key to combatting the HIV/AIDS pandemic.

## Supplementary material

10.1136/bmjopen-2025-102999 online supplemental file 1

## Data Availability

Data are available in a public, open access repository.
