# Supplementary material for: Comparison of HIV self-test distribution modalities to reduce HIV transmission and burden in western Kenya: a mathematical modelling study
Source: BMJ Open. 2025 Jul 30;15(7):e102999. doi: 10.1136/bmjopen-2025-102999 (PMC12314966; doi:10.1136/bmjopen-2025-102999)
Supplement: online supplemental file 1 [file bmjopen-15-7-s001.docx]

**Supplement Material**

Title: Comparison of HIV self-test distribution modalities to reduce HIV transmission and burden in western Kenya: a mathematical modeling study


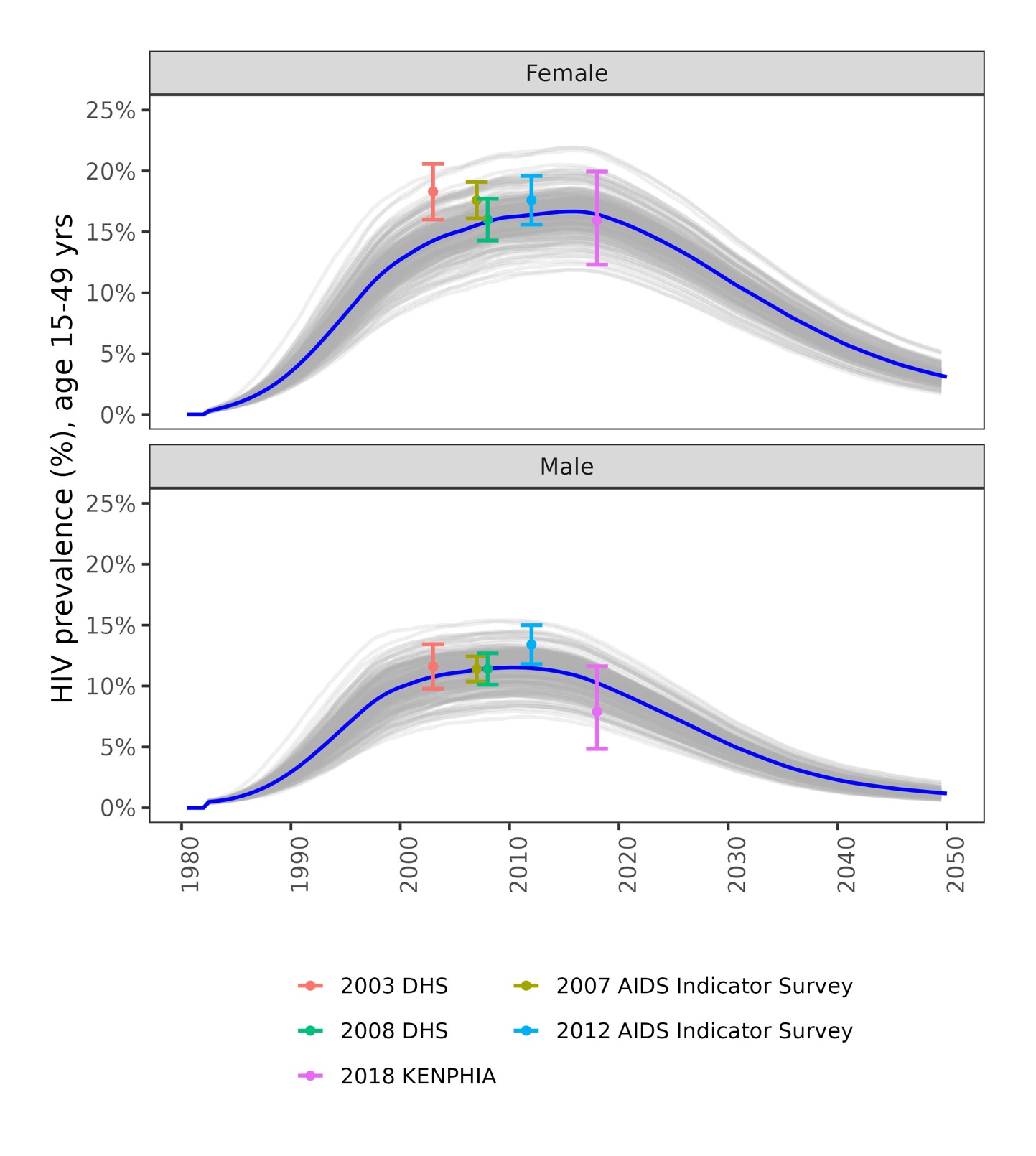


**Figure S1**. HIV prevalence among individuals aged 15-49 years in western Kenya (1983-2050) by gender. The blue line represents the mean modeled trajectory, the gray lines indicate 250 individual simulations, and the colored points with error bars represent observed data of HIV prevalence from various surveys. Acronyms: DHS, Demographic Health Survey; KENPHIA, Kenya Population-based HIV Impact Assessment

**
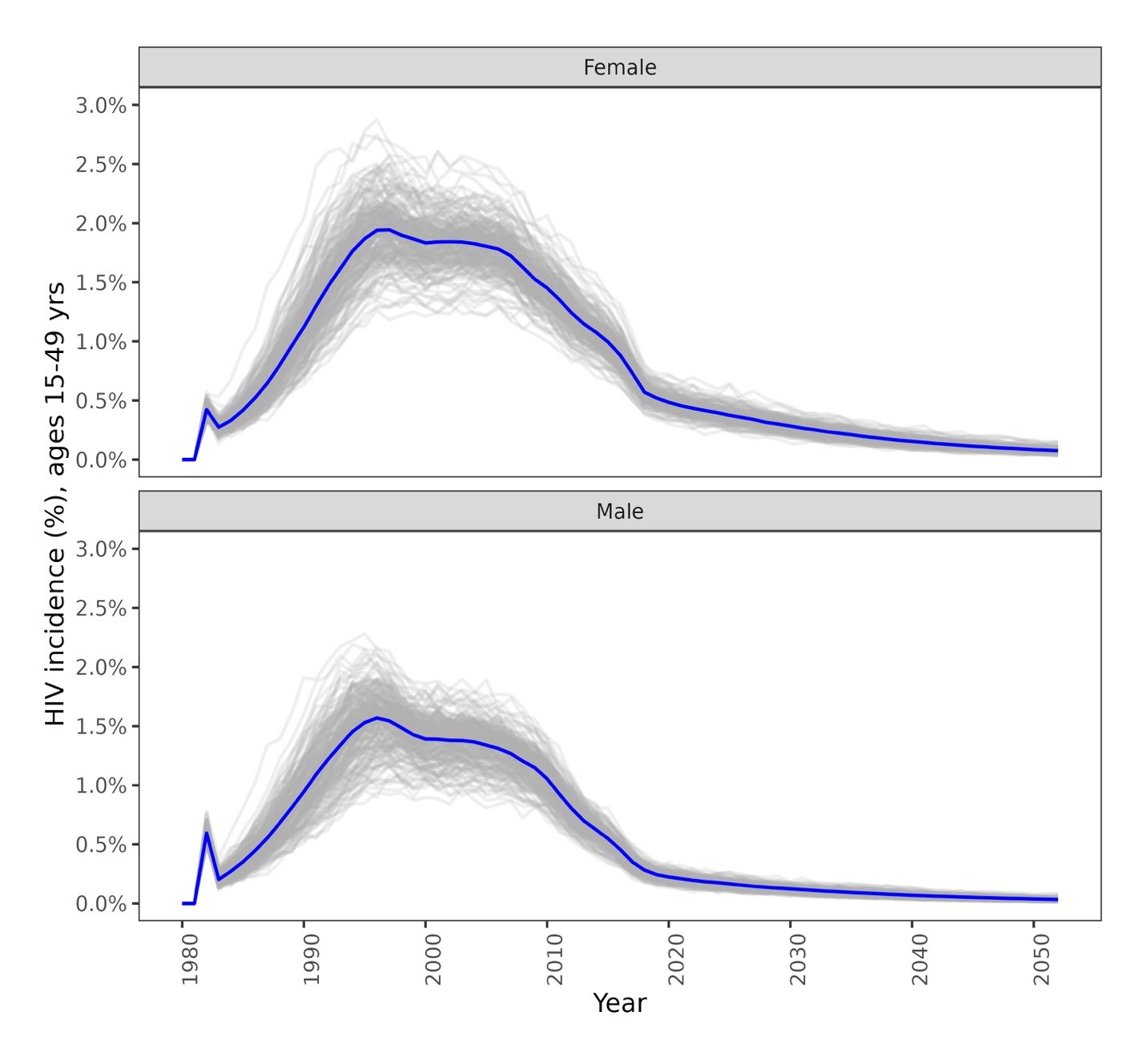
**

**Figure S2.** HIV incidence among individuals aged 15-49 years in western Kenya (1980-2050) by sex. The blue line represents the mean modeled trajectory, and the gray lines indicate 250 individual simulations.

**
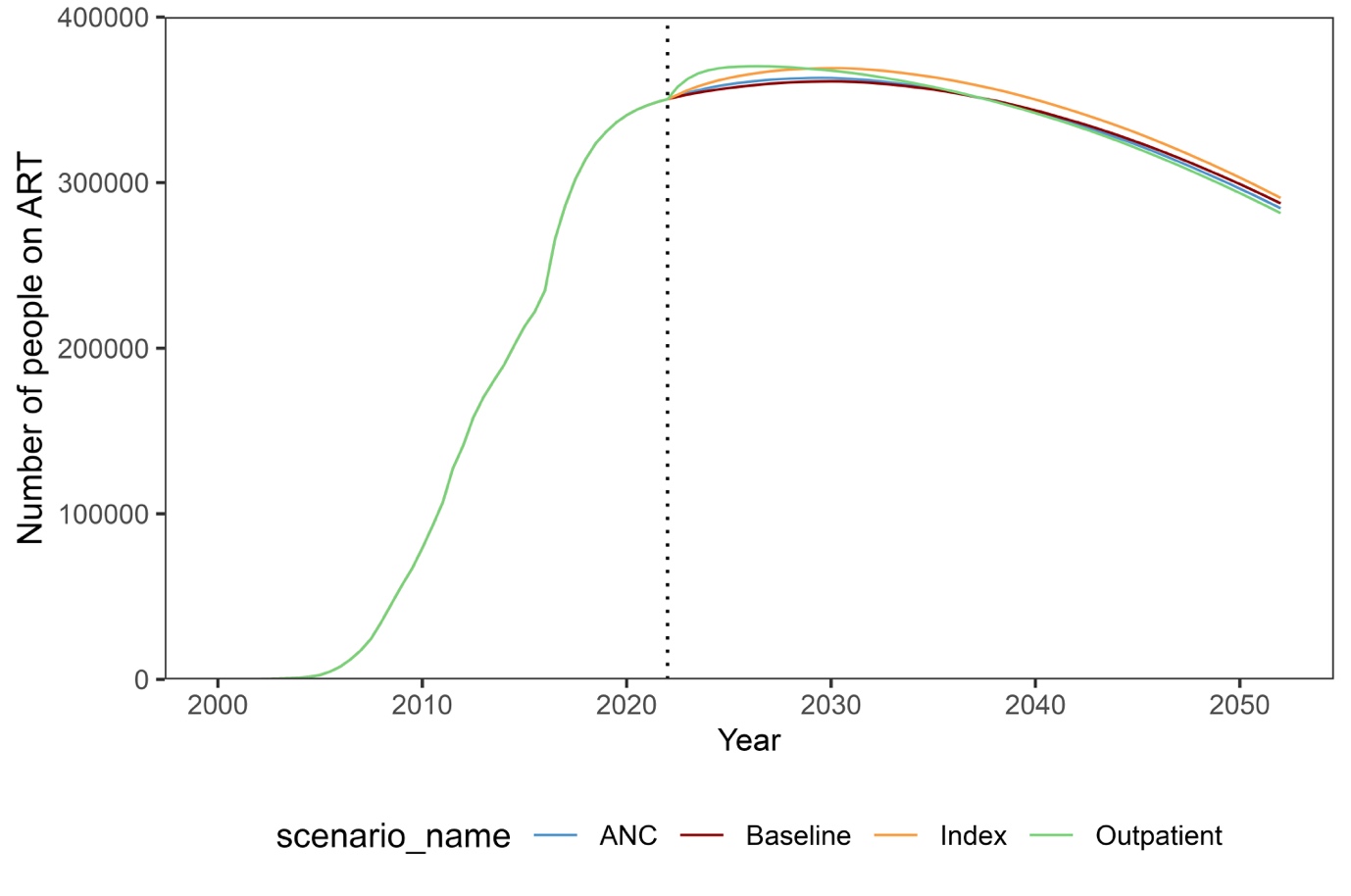
**

Outpatient

Partner services

ANC

SoC testing only

**Figure S3.** Number of people on ART by HIVST distribution modalities. The dotted line indicates the year when the simulation starts (2022).

**Table S1. Estimated number of HIV tests and outcomes by HIVST distribution modality and sex**

|  | **SoC** | | **Scenario 1: ANC** | | **Scenario 2: Partner services** | | **Scenario 3: Outpatient** | |
| --- | --- | --- | --- | --- | --- | --- | --- | --- |
|  | **Male** | **Female** | **Male** | **Female** | **Male** | **Female** | **Male** | **Female** |
| **HIV tests per year (1000s)** | 2,292 (2,290, 2,295) | 2,395 (2,392, 2,399) | 2,424 (2,421, 2,427) | 2,400 (2,397, 2,404) | 2,309 (2,307, 2,312) | 2,408 (2,404, 2,412) | 2,878 (2,876, 2,881) | 2,922 (2,919, 2,926) |
| Non-HIVST (1000s) | 2,292 (2,290, 2,295) | 2,395 (2,392, 2,399) | 2,338 (2,335, 2,341) | 2,400 (2,397, 2,404) | 2,303 (2,300, 2,305) | 2,403 (2,400, 2,407) | 2,291 (2,290, 2,292) | 2,395 (2,392, 2,397) |
| HIVST (1000s) | - | - | 86.0 (85.6, 86.3) | - | 6.9 (6.8, 7) | 4.5 (4.4, 4.6) | 587.0 (585.8, 588.1) | 527.9 (526.9, 528.9) |
| **HIV infections** |  |  |  |  |  |  |  |  |
| New HIV infections per year (1000s) | 2.3 (2.2, 2.3) | 4.3 (4.2, 4.3) | 2.2 (2.1, 2.2) | 4.0 (3.9, 4.0) | 2.0 (2.0, 2.1) | 3.8 (3.8, 3.9) | 2.1 (2.0, 2.1) | 3.9 (3.8, 4.0) |
| Cumulative new HIV infections (1000s) | 68.1 (66.7, 69.4) | 127.5 (125.2, 129.7) | 65.1 (63.8, 66.4) | 118.6 (116.5, 120.6) | 60.9 (59.7, 62.1) | 114.6 (112.6, 116.5) | 62.3 (61, 63.5) | 116.7 (114.6, 118.7) |
| **HIV-related deaths** |  |  |  |  |  |  |  |  |
| HIV-related deaths per year (1000s) | 3.0 (3.0, 3.1) | 4.5 (4.4, 4.5) | 2.8 (2.8, 2.9) | 4.4 (4.4, 4.5) | 2.7 (2.6, 2.7) | 4.1 (4.1, 4.2) | 2.9 (2.8, 2.9) | 4.3 (4.3, 4.4) |
| Cumulative HIV-related deaths (1000s) | 90.6 (89.2, 91.9) | 133.8 (132.2, 135.5) | 84.9 (83.6, 86.1) | 132.4 (130.8, 134) | 79.6 (78.4, 80.7) | 124.4 (122.9, 125.8) | 85.9 (84.6, 87.2) | 129.8 (128.2, 131.4) |
| **ART** |  |  |  |  |  |  |  |  |
| Number of people on ART per year (1000s) | 120.0 (118.1, 121.8) | 221.1 (218.2, 223.9) | 123.7 (121.8, 125.6) | 217.3 (214.5, 220.1) | 125.2 (123.3, 127.1) | 221.9 (219, 224.7) | 122.8 (120.9, 124.6) | 219.9 (217.1, 222.8) |
| Cumulative person-years on ART (1000s) | 3,599 (3,544, 3,654) | 6,633 (6,546, 6,717) | 3,710 (3,653, 3,767) | 6,519 (6,435, 6,602) | 3,757 (3,700, 3,814) | 6,658 (6,571, 6,742) | 3,683 (3,626, 3,739) | 6,598 (6,512, 6,683) |

*****95% confidence intervals are shown in parentheses.

**Cumulative HIV infections, HIV-related deaths, and person-years on ART are over the 30 years of simulation period (2022 – 2051).

**Table S2. Estimated number of HIV tests and outcomes by HIVST distribution modality between 2022-2032**

|  | **SoC** | **Scenario 1: ANC** | **Scenario 2: Partner services** | **Scenario 3: Outpatient** |
| --- | --- | --- | --- | --- |
| **HIV tests per year (1000s)** | 3,404 (3,399, 3,408) | 3,507 (3,503, 3,512) | 3,425 (3,421, 3,430) | 4,504 (4,501, 4,508) |
| Non-HIVST (1000s) | 3,404 (3,399, 3,408) | 3,434 (3,429, 3,438) | 3,410 (3,406, 3,414) | 3,389 (3,388, 3,391) |
| HIVST (1000s) |  | 73.5 (73.1, 73.9) | 15.3 (15.1, 15.5) | 1,115 (1,113, 1,117) |
| **HIV infections** |  |  |  |  |
| New HIV infections per year (1000s) | 8.4 (8.3, 8.6) | 8.0 (7.9, 8.1) | 7.8 (7.6, 7.9) | 7.6 (7.5, 7.7) |
| Cumulative new HIV infections (1000s) | 84.3 (83, 85.5) | 80 (78.8, 81.2) | 77.6 (76.5, 78.7) | 76.2 (75.1, 77.4) |
| **HIV-related deaths** |  |  |  |  |
| HIV-related deaths per year (1000s) | 7.2 (7.1, 7.3) | 7.1 (7, 7.2) | 6.8 (6.7, 6.9) | 6.9 (6.8, 7) |
| Cumulative HIV-related deaths (1000s) | 72.1 (71.2, 73) | 70.7 (69.9, 71.6) | 68 (67.2, 68.8) | 68.9 (68.1, 69.7) |
| **ART** |  |  |  |  |
| Number of people on ART per year (1000s) | 358.4 (353.9, 362.8) | 360.2 (355.7, 364.7) | 364.7 (360.1, 369.2) | 367.4 (362.7, 371.9) |
| Cumulative person-years on ART (1000s) | 3,584 (3,539, 3,628) | 3,602 (3,557, 3,647) | 3,647 (3,601, 3,692) | 3,674 (3,627, 3,719) |

*****95% confidence intervals are shown in parentheses.

**Cumulative HIV infections, HIV-related deaths, and person-years on ART are over the 10 years of simulation period (2022 – 2031).

**Table S3. Estimated number of HIV tests and outcomes by HIVST distribution modality and sex between 2022-2032**

|  | **SoC** | | **Scenario 1: ANC** | | **Scenario 2: Partner services** | | **Scenario 3: Outpatient** | |
| --- | --- | --- | --- | --- | --- | --- | --- | --- |
|  | **Male** | **Female** | **Male** | **Female** | **Male** | **Female** | **Male** | **Female** |
| **HIV tests per year (1000s)** | 1,653 (1,651, 1,656) | 1,751 (1,747, 1,754) | 1,755 (1,752, 1,758) | 1,752 (1,748, 1,756) | 1,666 (1,664, 1,669) | 1,759 (1,755, 1,762) | 2,235 (2,233, 2,237) | 2,270 (2,266, 2,273) |
| Non-HIVST (1000s) | 1,653 (1,651, 1,656) | 1,751 (1,747, 1,754) | 1,682 (1,679, 1,685) | 1,752 (1,748, 1,756) | 1,657 (1,655, 1,660) | 1,753 (1,749, 1,756) | 1,648 (1,647, 1,649) | 1,742 (1,740, 1,744) |
| HIVST (1000s) |  |  | 73.5 (73.1, 73.9) |  | 9.3 (9.2, 9.4) | 6.0 (5.9, 6.1) | 587.0 (585.9, 588.2) | 527.9 (526.9, 528.9) |
| **HIV infections** |  |  |  |  |  |  |  |  |
| New HIV infections per year (1000s) | 2.8 (2.8, 2.9) | 5.6 (5.5, 5.7) | 2.8 (2.7, 2.8) | 5.2 (5.1, 5.3) | 2.6 (2.6, 2.7) | 5.1 (5.1, 5.2) | 2.6 (2.6, 2.6) | 5.0 (4.9, 5.1) |
| Cumulative new HIV infections (1000s) | 28.3 (27.8, 28.7) | 56 (55.2, 56.8) | 27.7 (27.3, 28.2) | 52.3 (51.5, 53) | 26.3 (25.8, 26.7) | 51.3 (50.6, 52.1) | 26 (25.5, 26.4) | 50.2 (49.5, 51) |
| **HIV-related deaths** |  |  |  |  |  |  |  |  |
| HIV-related deaths per year (1000s) | 3.3 (3.3, 3.3) | 3.9 (3.9, 4) | 3.2 (3.1, 3.2) | 3.9 (3.9, 3.9) | 3.1 (3, 3.1) | 3.7 (3.7, 3.8) | 3.1 (3.1, 3.2) | 3.8 (3.7, 3.8) |
| Cumulative HIV-related deaths (1000s) | 33 (32.5, 33.5) | 39.1 (38.6, 39.6) | 31.7 (31.3, 32.1) | 39 (38.6, 39.5) | 30.7 (30.3, 31.1) | 37.3 (36.9, 37.7) | 31.1 (30.7, 31.5) | 37.8 (37.3, 38.2) |
| **ART** |  |  |  |  |  |  |  |  |
| Number of people on ART per year (1000s) | 130.4 (128.5, 132.3) | 228 (225.2, 230.7) | 133.4 (131.5, 135.3) | 226.9 (224.1, 229.6) | 134.8 (132.8, 136.7) | 230 (227.2, 232.7) | 136.3 (134.3, 138.2) | 231.1 (228.3, 233.8) |
| Cumulative person-years on ART (1000s) | 1,304 (1,285, 1,323) | 2,280 (2,252, 2,307) | 1,334 (1,315, 1,353) | 2,269 (2,241, 2,296) | 1,348 (1,328, 1,367) | 2,300 (2,272, 2,327) | 1,363 (1,343, 1,382) | 2,311 (2,283, 2,338) |

*****95% confidence intervals are shown in parentheses.

**Cumulative HIV infections, HIV-related deaths, and person-years on ART are over the 10 years of simulation period (2022 – 2031).
