# Supplementary material for: Comparison of HIV self-test distribution modalities to reduce HIV transmission and burden in western Kenya: a mathematical modelling study
Source: BMJ Open. 2025 Jul 30;15(7):e102999. doi: 10.1136/bmjopen-2025-102999 (PMC12314966; doi:10.1136/bmjopen-2025-102999)
Supplement: Supplementary file 2 [file bmjopen-2025-102999.reviewer_comments.pdf]

PEER REVIEW HISTORY

BMJ Open publishes all reviews undertaken for accepted manuscripts. Reviewers are asked to complete a checklist review form and are provided with free text boxes to elaborate on their assessment. These free text comments are reproduced below.

ARTICLE DETAILS

Title (Provisional)

Comparison of HIV self-test distribution modalities to reduce HIV transmission and burden in western Kenya: a mathematical modeling study

Authors

Kim, Hae-Young; Eshun-Wilson, Ingrid; Bridenbecker, Daniel; Johnson, Cheryl; Kisia, Christine; Magare, Jonah Onetiah; Geng, Elvin; Bershteyn, Anna

---

VERSION 1 - REVIEW

**Reviewer**	**1**
**Name**	**Hamilton, Deven T.**
**Affiliation**	**University of Washington**
**Date**	**19-Aug-2024**
**COI**	**None**

This paper reports on findings from a simulation study estimating epidemic outcomes following the introduction of three alternative HIVST programs in Western Kenya. Overall, the research question is important and timely and the methods are appropriate. The model is not described in its entirety due to the complexity of the model and the availability of other manuscripts describing the model in more detail. The key finding is that the "index" modality is the most efficient and averted the largest number of new infections. I have few suggested revisions.

1) Even though EMOD is described elsewhere it would be helpful to have a visual point of reference to better understand what the model is predicting. The Model was calibrated to HIV prevalence in 2003 and 2008-9 and HIV was introduced in 1982. It would be helpful to see a plot of HIV prevalence and incidence from 1983 through the end or your simulation period (2052) for your reference model and under each of your intervention scenarios.
2) Please include the sensitivity, specificity and test windows for all tests used in the simulation and a discussion of their implications for your findings.
3) Please describe in more detail how individuals came to test for HIV in the baseline model and how testing changed when HIVST was introduced. In lines 128 through 137 you say that there is voluntary provider-initiated testing but you don't indicate how that is modeled. Is it a memoryless stochastic process or a fixed interval? Does testing depend on age, sex partnership status? There are some citations for studies that report different test coverage rates in different sub-populations but it is not clear how those reported levels of ever having been tested were used in the model. If someone used an HIVST how did that change the probability they would be tested in a clinic or some other setting in the next month or year.
4) The HIV treatment and prevention landscape has changed dramatically over the last 30 years with new ARVs, U=U, PrEP, injectable prep rapid test, NAAT tests etc. Given the rate of change in the epidemic landscape please provide a justification for projecting the impact of your HIVST interventions

out to 30 years. A shorted time period, perhaps a decade, would be more useful for making policy recommendations in the near term.

**Reviewer**	**2**
**Name**	**Woldegerima, Woldegebriel Assefa**
**Affiliation**	**York University, Mathematics and Statistics**
**Date**	**19-Aug-2024**
**COI**	**None**

Dr. Woldegebriel Assefa Woldegerima, York University
Comments to the Author:
The research objectives and methods are interesting, and applying an IBM using the publicly existing EMOD-HIV model is an important approach to estimating or evaluating the impact of intervention strategies such as testing, treatment, etc.
1) However, when simulating the impact of three HIV self-testing (HIVST) distribution modalities, have you also considered the sensitivity of your model to geographic locations within Western Kenya? What about the awareness of individuals towards self-testing?
2) In least-developed countries, such as rural areas of Kenya, the availability and distribution of testing kits face several economic, environmental, and self-awareness factors. Particularly, individual behaviour and awareness can limit the effectiveness of your proposed testing approach. Have you considered this in your model or the limitations of your results?
3) What was the computation time required to run the EMOD-HIV model in your case? How many times have you run the model? I am asking this question since this may impact or alter your concluding results due to implementation efficiency and difficulty of EMOD?
4) Why was confidence interval not used or stated in most results? For example, under the result's paragraph of the abstract: you specifically mentioned that "ndex modality will avert the largest number of new HIV infections (10.3%), followed by facility (9.8%) and ANC (4.6%)....". My concern is those numbers seem too specific and unrealistic. This may depend on the noise of the data; the number of times the EMOD setting is run, and most importantly, EMOD is typically useful and provides better results when applied in big data. How big was your data?
Additionally, since you used IBM (ABM), I expected some sort of probabilistic results with a confidence interval.
The same comment goes to the next lines, where you stated "Compared to SoC, the number of HIVST needed per additional HIV infection averted was 173 with ANC....".

5). On page 10 of 22, lines 295-297, you used reference 43, i.e., Ortblad et al. (2018) to justify your limitation of not considering PrEP. You mentioned, "Studies are ongoing to understand the role of HIVST for PrEP initiation and continuation".
However, a recent study by Cox et al. (2024), titled " Impact of HIV self-testing for oral pre-exposure prophylaxis scale-up on drug resistance and HIV outcomes in western Kenya: a modeling study" has investigated the impact of HIST on PrEP. They clearly concluded that " Community-based oral pre-exposure prophylaxis (PrEP) provision has the potential to expand PrEP coverage. HIV self-testing can facilitate PrEP community-based delivery but might have lower sensitivity than facility-based HIV testing, potentially leading to inappropriate PrEP use among people with HIV and subsequent development of drug resistance". So, how do you align your study and your results with this recent study?
6) I would also suggest the authors to proofread the whole manuscript.
In my opinion, if the authors revised these a few comments and other comments provided by other reviewers, this manuscript is publishable in BMJ.

VERSION 1 - AUTHOR RESPONSE

Reviewer: 1
Dr. Deven T. Hamilton, University of Washington

We sincerely thank you for providing thorough and insightful comments.

Comments to the Author:
This paper reports on findings from a simulation study estimating epidemic outcomes following the introduction of three alternative HIVST programs in Western Kenya. Overall, the research question is important and timely and the methods are appropriate. The model is not described in its entirety due to the complexity of the model and the availability of other manuscripts describing the model in more detail. The key finding is that the "index" modality is the most efficient and averted the largest number of new infections. I have few suggested revisions.

1) Even though EMOD is described elsewhere it would be helpful to have a visual point of reference to better understand what the model is predicting. The Model was calibrated to HIV prevalence in 2003 and 2008-9 and HIV was introduced in 1982. It would be helpful to see a plot of HIV prevalence and incidence from 1983 through the end or your simulation period (2052) for your reference model and under each of your intervention scenarios.

Thank you for the suggestion. We have now included calibration plots for HIV prevalence (Figure S1) and HIV incidence (Figure S2) in the supplement.

2) Please include the sensitivity, specificity and test windows for all tests used in the simulation and a discussion of their implications for your findings.

Thank you for this point. According to the WHO guideline, HIV diagnosis is based on three consecutive reactive assays of approved rapid HIV antibody testing kits, which have shown 99.6% sensitivity and 100% specificity. We therefore assumed both sensitivity and specificity of 100%.[1]

In addition, Determine HIV 1/2 test (Abbott Diagnostic Medical Co.Ltd), which is used for HIVST in Kenya,[23] has sensitivity of 99.9% (95% CI: 99.4%, 100%) and specificity of 100% (95% CI: 98.0%, 100%) in a controlled environment.[4] In a systematic review and meta-analysis which looked at reliability of HIV rapid diagnostic for self-testing compared with testing by healthcare workers found that the pooled agreement (Kappa) for unobserved HIVST and unassisted HIVST with a HIV test conducted by a healthcare worker was 0.96 (95% CI: 0.94, 0.99), and 0.97 (95% CI: 0.96, 0.98).[5] Also, all individuals who are linked to care after HIVST undergo a confirmatory testing using the national testing algorithm (i.e., serial testing). Therefore, we assumed that sensitivity and specific of HIVST are also 100%. We have added the following line in the manuscript:

Line 176: *"We assumed that the sensitivity and specificity of rapid HIV tests are both 100%."*

3) Please describe in more detail how individuals came to test for HIV in the baseline model and how testing changed when HIVST was introduced. In lines 128 through 137 you say that there is voluntary provider-initiated testing but you don't indicate how that is modeled. Is it a memoryless stochastic process or a fixed interval? Does testing depend on age, sex partnership status? There are some citations for studies that report different test coverage rates in different sub-populations but it is not clear how those reported levels of ever having been tested were used in the model. If someone used an HIVST how did that change the probability they would be tested in a clinic or some other setting in the next month or year.

Thank you for these comments. We assumed that uptake of HIV testing first occurs at sexual debut with the probability following a sigmoid function then is presented a chance to re-test

annually. For those who does not undergo HIV testing at sexual test, similarly, they receive a chance to choose an HIV test with the following a sigmoid function. If someone used HIVST and was tested HIV-negative, we considered that the person had received his or her annual HIV testing (i.e., replacing the rapid HIV test the person might have received at a health facility). We have added the following:

Line 124-132: "Within the model, baseline HIV testing modalities include voluntary or provider-initiated testing at healthcare facilities, testing of pregnant women during ANC visits, and symptomatic testing for individuals who progress to advanced HIV disease. For voluntary or provider-initiated testing at health facilities, individuals uptake HIV testing upon their sexual debut, with the probability following a sigmoid function that varies by calendar year. Individuals who do not undergo HIV testing at sexual debut are assigned a random probability of testing once a year thereafter. After an individual completes his or her initial HIV test, the frequency of repeat HIV testing remains annual, with a random, exponentially distributed time between repeat tests."

Line 156-177: "For voluntary or provider-initiated testing at health facilities, we assumed that the probability of HIV testing uptake at sexual debut follows a sigmoid function, increasing from 0% to 5% with an inflection point in 2005. The probability of HIV testing uptake for annual repeat-testing after sexual debut is also assumed to increase sigmoidally from 0% to 15%, with an inflection point in 2006, and then continue to increase based on the reported data on annual HIV testing and overall HIV testing history. In our model simulations, the average percentage of adults aged >15 years who report having ever tested for HIV was 80.3% in 2018, increasing to 82.1% in 2021. This is comparable to the reported figure of 77.9% from the KENPHIA 2018 survey.2 Additionally, our simulations estimated that the average percentage of women and men aged 15-49 who received an HIV in the past 12 months was 47.4% in XX, which was comparable to the reported figures of 52.8% for women and 45.7% for men in 2014 (DHS), and 53.1% for women and 36.4% in men in 2018 (KENPHIA).

The probability of HIV testing at 12 weeks of pregnancy during ANC visits was assumed to increase sigmoidally from 0% in 1990 to 98% in 2011 based on the Kenya AIDS indicator surveys, in which 97.3% of women aged 15-49 years who delivered reported attending at least one ANC visit and of these, 96.0% knew their HIV status.22829 Similarly, the probability of symptom-driven HIV testing was assumed to increase sigmoidally from 0% in 1990 to 95% in 2016. This assumption was based on a survey conducted in coastal Kenya from 2017 to 2019, which found that 93.1% of individuals who presented to clinics with symptoms of acute infectious illness and were offered HIV testing accepted it.32 Lastly, we assumed that the sensitivity and specificity of rapid HIV tests are both 100%."

4) The HIV treatment and prevention landscape has changed dramatically over the last 30 years with new ARVs, U=U, PrEP, injectable prep rapid test, NAAT tests etc. Given the rate of change in the epidemic landscape please provide a justification for projecting the impact of your HIVST interventions out to 30 years. A shorted time period, perhaps a decade, would be more useful for making policy recommendations in the near term.

Thank you for the commenets. As a sensitivity analysis, we have limited the analysis to 10 years and included it in the supplement materials.

Line 238-241: "*In one-way sensitivity analyses, we varied the effects of HIVST on uptake using the 95% confidence intervals of the effect estimates from the meta-analysis. Additionally, we limited the time horizon of the analysis to 10 years to assess the relatively short-term policy impact, as HIV testing and treatment methods may change over the next 30 years (Table S1)."*

Reviewer: 2
Dr. Woldegebriel Assefa Woldegerima, York University
Comments to the Author:
The research objectives and methods are interesting, and applying an IBM using the publicly existing EMOD-HIV model is an important approach to estimating or evaluating the impact of intervention strategies such as testing, treatment, etc.

1) However, when simulating the impact of three HIV self-testing (HIVST) distribution modalities, have you also considered the sensitivity of your model to geographic locations within Western Kenya? What about the awareness of individuals towards self-testing?

Thank you for this comment. As described in the methods, the model was calibrated to HIV prevalence, number of people on ART, and population size, all of which were stratified by six countries as well as age and gender. The uptake of HIVST remains low across all six counties and nationwide. As mentioned in the introduction, of adults who were ever tested for HIV, only 4% reported having ever taken an HIV self-test in Kenya. Therefore, we did not differentiate in terms of awareness and uptake of HIVST by counties.

2) In least-developed countries, such as rural areas of Kenya, the availability and distribution of testing kits face several economic, environmental, and self-awareness factors. Particularly, individual behaviour and awareness can limit the effectiveness of your proposed testing approach. Have you considered this in your model or the limitations of your results?

Thank you for this comment. We agree that many individual, economic, and environmental factors can play a significant role in the uptake of HIVST. In our model, we assumed the effects of HIVST on uptake and linkage to care were derived from the meta-analysis of randomized clinical trials thus could be different from real-world implementation. We have added this as a limitation as following:

Line 368-371*: "Also, the scale-up of HIVST distribution can be complex and depends on many other factors, such as supply chain, healthcare infrastructure, and public awareness, which have not been explicitly modelled in our analyses."*

3) What was the computation time required to run the EMOD-HIV model in your case? How many times have you run the model? I am asking this question since this may impact or alter your concluding results due to implementation efficiency and difficulty of EMOD?

Thank you for these comments. We have selected 250 best-fit trajectories after calibration; each simulation took about 1hr using parallel high-performing computing system at NYULH. We have added more details about the number of simulations and computation time as following:

Line 151-152: *"We selected a set of 250 best-fit model trajectories using roulette resampling in proportion to likelihood."*

4) Why was confidence interval not used or stated in most results? For example, under the result's paragraph of the abstract: you specifically mentioned that "ndex modality will avert the largest number of new HIV infections (10.3%), followed by facility (9.8%) and ANC (4.6%)....". My concern is those numbers seem too specific and unrealistic. This may depend on the noise of the data; the number of times the EMOD setting is run, and most

importantly, EMOD is typically useful and provides better results when applied in big data. How big was your data?

Additionally, since you used IBM (ABM), I expected some sort of probabilistic results with a confidence interval. The same comment goes to the next lines, where you stated “Compared to SoC, the number of HIVST needed per additional HIV infection averted was 173 with ANC....”.

Thank you for these comments. We have now added 95% CI intervals for all reported model results using 10,000 bootstrap samples and added the following in the Methods:

Line 232-235: “*The reported results represent the means of 250 best-fit model trajectories derived from the calibration process. The 95% confidence intervals were estimated using 10,000 bootstrap samples generated using R package boot.”*

5). On page 10 of 22, lines 295-297, you used reference 43, i.e., Ortblad et al. (2018) to justify your limitation of not considering PrEP. You mentioned, "Studies are ongoing to understand the role of HIVST for PrEP initiation and continuation".

However, a recent study by Cox et al. (2024), titled " Impact of HIV self-testing for oral pre-exposure prophylaxis scale-up on drug resistance and HIV outcomes in western Kenya: a modeling study" has investigated the impact of HIST on PrEP. They clearly concluded that " Community-based oral pre-exposure prophylaxis (PrEP) provision has the potential to expand PrEP coverage.

HIV self-testing can facilitate PrEP community-based delivery but might have lower sensitivity than facility-based HIV testing, potentially leading to inappropriate PrEP use among people with HIV and subsequent development of drug resistance". So, how do you align your study and your results with this recent study?

Thank you for these comments. As you know, WHO recently issued a guideline to recommend PrEP initiation. We have discussed this in-depth with WHO and now included one of the experts who developed the guideline as a co-author (Dr Cheryl Johnson). Based on these comments and available evidence, we have incorporated these and updated our discussion section.

As of today, this recommendation has not been implemented in a real-world/routine practice setting/implemented only in small pilot projects. We plan to revisit this question as we have more data. We now have added this in the limitation and as a future direction in discussion as following:

Line 361-371: *“Our study has several limitations. First, we did not consider potential services for individuals with a negative self-test, such as accessing PEP or initiation or continuation of PrEP. Second, we did not evaluate the costs or cost-effectiveness of HIVST, as implementation costs were not available for all distribution modalities. A recent modelling study in South Africa reported that secondary distribution to partners of ART patients had the greatest epidemiological impact but was the least cost-effective strategy, while distribution of HIVST at workplaces would be cost-saving but have a moderate impact on averting HIV infections.54 In the context of PrEP, a recent study showed using HIVST over standard testing in Kenya was more affordable and less costly.55 Also, the scale-up of HIVST distribution can be complex and depends on many other factors, such as supply chain, healthcare infrastructure, and public awareness, which have not been explicitly modelled in our analyses.”*

Line 373-382: *"Looking ahead, it is critical to evaluate the role of HIVST in addressing gaps in delivery of timely HIV prevention services and diagnosis, while achieving more efficient benefits. Expanding the integration of HIVST and delivery systems beyond traditional HIV service points - such as worksites, private pharmacies, and mobile platforms, could further enhance accessibility, uptake and costs. HIVST could also facilitate earlier diagnoses when offered a sufficiently high volume. While most cost evaluation or cost-effectiveness analyses of HIVST have been conducted from a health system perspective, assessing cost-effectiveness from both end-user and societal perspectives will be valuable. Lastly, future studies could consider budget impact analysis to determine the proportion of HIVST within overall HIV programs and health system budgets, offering insights into the implications of incorporating HIVST for optimal resource allocation."*

6) I would also suggest the authors to proofread the whole manuscript.

Thank you for the suggestion. We have read our manuscript carefully and made edits throughout.

In my opinion, if the authors revised these a few comments and other comments provided by other reviewers, this manuscript is publishable in BMJ.

Thank you.

VERSION 2 - REVIEW

**Reviewer**	**1**
**Name**	**Hamilton, Deven T.**
**Affiliation**	**University of Washington**
**Date**	**15-Apr-2025**
**COI**	**None**

The authors have addressed all of the concerns I had with the initial submission.

**Reviewer**	**2**
**Name**	**Woldegerima, Woldegebriel Assefa**
**Affiliation**	**York University, Mathematics and Statistics**
**Date**	**21-Apr-2025**
**COI**	**None**

The authors addressed all comments, and revised the manuscript accordingly.
